# Supplementary material for: The interactome of intact mitochondria by cross-linking mass spectrometry provides evidence for coexisting respiratory supercomplexes
Source: Mol Cell Proteomics. 2017 Dec 8;17(2):216–32. doi: 10.1074/mcp.RA117.000470 (PMC5795388; doi:10.1074/mcp.RA117.000470)
Supplement: Supplemental Data [file supp_RA117.000470_133922_0_supp_23978_fzffwf.zip › spectra_annotation/mito_spectra_annotation/364-1-10-1-3-1.pdf]

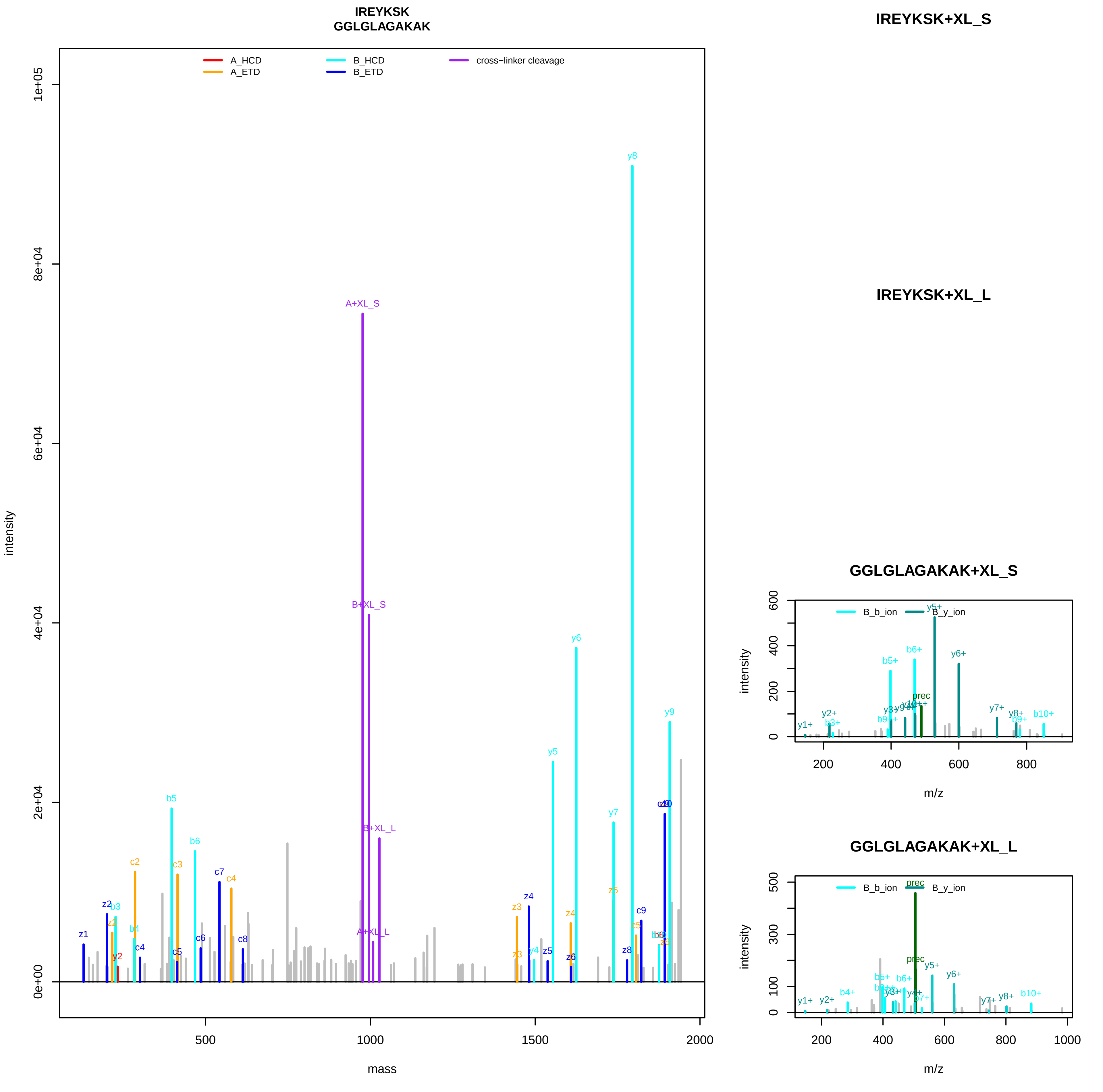
IREYKSK
GGLGLAGAKAK
A_HCD
A_ETD
B_HCD
B_ETD
cross−linker cleavage
intensity
mass
0e+00
2e+04
4e+04
6e+04
8e+04
1e+05
500
1000
1500
2000
A+XL_S
B+XL_S
B+XL_L
A+XL_L
y8
y6
y9
y5
y7
b5
b6
c2
c3
c4
c7
c6
c5
c8
c9
c10
z1
z2
z3
z4
z5
z6
z8
b3
b4
c4
y2
y4
IREYKSK+XL_S
IREYKSK+XL_L
GGLGLAGAKAK+XL_S
B_b_ion
B_y_ion
prec
m/z
GGLGLAGAKAK+XL_L
